# Pre- versus post-operative untargeted plasma nuclear magnetic resonance spectroscopy metabolomics of pheochromocytoma and paraganglioma

**DOI:** 10.1007/s12020-021-02858-z

**Published:** 2021-09-18

**Authors:** Nikolaos G. Bliziotis, Leo A. J. Kluijtmans, Sebastian Soto, Gerjen H. Tinnevelt, Katharina Langton, Mercedes Robledo, Christina Pamporaki, Udo F. H. Engelke, Zoran Erlic, Jasper Engel, Timo Deutschbein, Svenja Nölting, Aleksander Prejbisz, Susan Richter, Cornelia Prehn, Jerzy Adamski, Andrzej Januszewicz, Martin Reincke, Martin Fassnacht, Graeme Eisenhofer, Felix Beuschlein, Matthias Kroiss, Ron A. Wevers, Jeroen J. Jansen, Jaap Deinum, Henri J. L. M. Timmers

**Affiliations:** 1 Department of Laboratory Medicine, Translational Metabolic Laboratory, Radboud University Medical Center, Nijmegen, the Netherlands; 2 Department of Analytical Chemistry, Institute for Molecules and Materials, Radboud University, Nijmegen, the Netherlands; 3 Department of Medicine III, University Hospital Carl Gustav Carus, Technische Universität Dresden, Dresden, Germany; 4 Hereditary Endocrine Cancer Group, Spanish National Cancer Research Centre (CNIO), Madrid, Spain and Centro de Investigación Biomédica en Red de Enfermedades Raras (CIBERER), Madrid, Spain; 5 Klinik für Endokrinologie, Diabetologie und Klinische Ernährung, Universitätsspital Zürich, Zürich, Switzerland; 6 Biometris, Wageningen UR, Wageningen, The Netherlands; 7 Schwerpunkt Endokrinologie/Diabetologie, Medizinische Klinik und Poliklinik I, Universitätsklinikum Würzburg, Zürich, Germany; 8 Medicover Oldenburg MVZ, Oldenburg, Germany; 9 Medizinische Klinik und Poliklinik IV, Klinikum der Universität, Ludwig-Maximilians-Universität, München, Munich, Germany; 10 Department of Hypertension, Institute of Cardiology, Warsaw, Poland; 11 Institut für Klinische Chemie und Labormedizin, Universitätsklinikum Carl Gustav Carus, Dresden, Germany; 12 Helmholtz Zentrum München, Research Unit Molecular Endocrinology and Metabolism, Neuherberg, Germany; 13 Institute of Biochemistry, Faculty of Medicine, University of Ljubljana, Ljubljana, Slovenia; 14 Institute of Experimental Genetics, Helmholtz Zentrum München, German Research Center for Environmental Health, Neuherberg, Germany; 15 Department of Biochemistry, Yong Loo Lin School of Medicine, National University of Singapore, Singapore, Singapore; 16 Core Unit Clinical Mass Spectrometry, University Hospital Würzburg, Würzburg, Germany; 17 Comprehensive Cancer Center Mainfranken, Universität Würzburg, Würzburg, Germany; 18 Department of Internal Medicine, Radboud University Medical Center, Nijmegen, the Netherlands

**Keywords:** PPGL, Metabolomics, NMR, Paired, Plasma, Operation

## Abstract

**Purpose:**

Pheochromocytomas and Paragangliomas (PPGL) result in chronic catecholamine excess and serious health complications. A recent study obtained a metabolic signature in plasma from PPGL patients; however, its targeted nature may have generated an incomplete picture and a broader approach could provide additional insights. We aimed to characterize the plasma metabolome of PPGL patients before and after surgery, using an untargeted approach, and to broaden the scope of the investigated metabolic impact of these tumors.

**Design:**

A cohort of 36 PPGL patients was investigated. Blood plasma samples were collected before and after surgical tumor removal, in association with clinical and tumor characteristics.

**Methods:**

Plasma samples were analyzed using untargeted nuclear magnetic resonance (NMR) spectroscopy metabolomics. The data were evaluated using a combination of uni- and multi-variate statistical methods.

**Results:**

Before surgery, patients with a nonadrenergic tumor could be distinguished from those with an adrenergic tumor based on their metabolic profiles. Tyrosine levels were significantly higher in patients with high compared to those with low BMI. Comparing subgroups of pre-operative samples with their post-operative counterparts, we found a metabolic signature that included ketone bodies, glucose, organic acids, methanol, dimethyl sulfone and amino acids. Three signals with unclear identities were found to be affected.

**Conclusions:**

Our study suggests that the pathways of glucose and ketone body homeostasis are affected in PPGL patients. BMI-related metabolite levels were also found to be altered, potentially linking muscle atrophy to PPGL. At baseline, patient metabolomes could be discriminated based on their catecholamine phenotype.

## Introduction

Pheochromocytomas and Paragangliomas (PPGL) are rare neuroendocrine tumors of neural crest-derived cells, which can be classified as adrenergic or nonadrenergic (noradrenergic and/or dopaminergic), depending on the profile of catecholamine metabolites [1]. Tumorigenesis can be driven by germline mutations in hereditary forms, as well as by somatic mutations [2]. PPGLs can also be classified in two main clusters based on their expression profiles. Cluster 1 is characterized by a pseudohypoxic response and includes mutations in VHL, succinate dehydrogenase (SDHx) and EPAS1, whereas cluster 2, is characterized by activation of the tyrosine kinase receptor and includes mutations in RET and NF1 [3]. Clusters can be distinguished based on tumoral catecholamine content, as well as secretory rates and catecholamine biochemical phenotype (adrenergic or nonadrenergic) [4]. Even so, a feature common in both PPGL clusters is overall catecholamine excess, which can lead to a vast array of symptoms, such as palpitations, headaches, profuse sweating and hypertension [5]. Patients may also present with metabolic alterations, particularly impaired glucose homeostasis [6].

Metabolomics is the study of all metabolite levels in a given biological fluid, also known as the metabolome [7]. Plasma has been used in metabolomics studies to investigate both rare diseases, such as inborn errors of metabolism [8], as well as more common diseases, such as cardiovascular impairment, diabetes mellitus, Parkinson’s disease and depression [9]. Analytical methods include liquid chromatography - mass spectrometry (LC-MS), as well as proton nuclear magnetic resonance spectroscopy (^1^H-NMR) [7]. LC-MS is known for its high sensitivity and large number of detectable metabolites, whereas NMR is a technique that is highly reproducible and provides increased possibilities for structure elucidation of unknown compounds [10]. As different sets of metabolites can be detected with each method, LC-MS and NMR metabolomics have been applied simultaneously on the same samples, to provide complementary results [11]. Depending on the research question, metabolomics can be applied in a targeted or untargeted fashion. Targeted metabolomics is better suited for when there is a predefined list of relevant metabolites, whereas untargeted metabolomics is preferred when the goal is to study the whole metabolome, including signals from unknown metabolites or metabolites of unknown relevance [12]. Recently, Erlic et al. [13] employed targeted LC-MS metabolomics for comparing metabolite levels in patients before and after surgical removal of PPGLs. The authors found significant alterations in amino acids, glycerophospholipids, sphingomyelins and a monosaccharide. These changes were linked to clinical features associated with PPGL, such as (pre-) diabetes mellitus, catecholamine-induced catabolic state, increased cardiovascular risk and post-operative weight increase.

In the present study, we applied untargeted NMR metabolomics [14] to plasma samples collected from patients before and after surgical removal of a PPGL. Our primary goal was to examine in an exploratory approach the impact of tumorous catecholamine excess on plasma metabolome, without predefining a set of potentially relevant compounds. NMR spectroscopy was selected as the analytical method, to focus on the strongest effects PPGL tumors have on patient plasma metabolome. We compared our results with those of Erlic et al. [13], thus taking advantage of the complementarity of the two approaches.

## Materials and methods

### Patients and samples

Samples were collected from 36 patients with biochemically and histologically proven PPGL according to the Prospective Monoamine-producing Tumor (PMT) study protocol [15]. For this international study, PPGL patients were diagnosed and treated at the following centers: Institute of Cardiology Warsaw Poland, University Hospital Dresden Germany, Radboud University Medical Center Nijmegen the Netherlands, Klinikum der Ludwig-Maximilians-Universität München Germany, University Hospital Würzburg Germany, University Medical Center Schleswig-Holstein Lübeck Germany. All patients were part of the Prospective Monoamine-producing Tumor (PMT) study, and sample collection took place according to a standardized protocol, in order to minimize effects of preanalytical biases [15]. In short, blood was drawn after an overnight fast and a 12 h-abstinence from alcohol, nicotine and caffeine. Patients maintained a fully supine position for 30 min before blood sampling (10 mL). Heparinized tubes were used to collect blood samples, which were subsequently placed on ice or cool pads. Plasma was collected after centrifugation at 4 °C, for 10 min at 3000 × *g* and stored at −80 °C until analysis. Samples were taken both before and after surgical removal of the tumor. No patients had evidence of metastasis, based on the absence of PPGL tissue in non-chromaffin organs. The tumoral catecholamine phenotype was determined as either adrenergic or nonadrenergic using plasma levels of metanephrines and the criteria described in Pamporaki et al. [16]. The study protocol was approved by local ethical committees and written informed consent was obtained from all participants.

As part of the standard work-up in the PMT study, patients were screened for the presence of mutations by means of the PheoSeq targeted gene panel [17], as described in a previous report [13]. All patients were tested for both somatic and germline mutations, except Warsaw patients (*n* = 18), for which only germline testing was carried out.

Both plasma free metanephrines and urine catecholamines were measured for all patients pre- and postoperatively according to the PMT study protocol using well-established methods [15].

### Untargeted ^1^H-NMR metabolomics

Plasma samples were prepared according to our previously described method [14] and were analyzed as part of a larger NMR metabolomics study. The PPGL study samples were randomized over the course of 48 batches, along with Quality Control (QC) and Healthy Volunteer (HV) samples, with the two latter groups only used for data processing before statistical analysis. The method was applied as described previously [14], with specific details listed in the supplementary information, section 2.2.

Untargeted as well as targeted peak identification was done by means of signal fitting, using Chenomx evaluation v. 8.4 [18] and Bruker Topspin v. 4.0.6. The human metabolome database [19], along with the Madison Metabolomics Consortium Database [20], were used as references for metabolite identification. On selected HV and QC samples, 2D NMR experiments were carried out to assign unknown peaks to a metabolite. Spiking experiments were done to confirm assignments (supplementary information, section 2.2). For biochemical pathway investigation, the Kyoto Encyclopedia of Genes and Genomes KEGG [21], was employed.

### Data analysis and statistics

Detailed information regarding statistical methods can be found in the supplementary information, section 2.3. In short, we explored the effects of clinical, biological and technical (preanalytical and analytical) factors on the metabolome, using both uni- and multi-variate analyses at baseline (including only measurements in pre-operative patient samples), as well as comparing pre- vs. post-operative in a paired manner, in accordance with a similar study [13]. Patients were grouped based on either binary factors or continuous covariates (using the median value as a cutoff for making two groups), to investigate the effect of each factor separately. Factors investigated include center of origin, sample age, patient sex, patient age, tumor size, total free plasma metanephrines (sum of concentrations of metanephrine, normetanephrine and methoxytyramine), total free urine catecholamines (sum of outputs of epinephrine, norepinephrine and dopamine), BMI, presence of hypertension, presence of diabetes mellitus, tumor location (adrenal/extra-adrenal), catecholamine biochemical (adrenergic/nonadrenergic), days before surgery (at baseline), days between pre- and post-operative sampling (for comparing pre-to postoperative), analytical batch, run order, presence of cluster 1 or 2 mutations, presence of detected mutations and presence of SDHx mutations. Multivariate methods were applied via the “MixOmics” [22] R package and include Principal Component Analysis (PCA), Partial Least Squares Discriminant Analysis (PLSDA), their paired equivalents [23], as well as classic Partial Least Squares (PLS) as a multivariate regression method. Peaks that were found to contribute to a multivariate model were deemed “important”. Univariate tests used to complement multivariate results included the Spearman correlation, Wilcoxon-tests for comparison of averages and the Shapiro-Wilkinson test for estimating data normality. For all *p* values generated, a false discovery rate correction [24] was used to account for multiple testing. A corrected *p* value of less than 0.05 was accepted as statistically significant.

## Results

### Patient and sample characteristics

The characteristics of the 36 patients included in the present study are summarized in Table 1. Thirty patients had a single adrenal tumor, one had bilateral adrenal tumors, four had a single extra-adrenal paraganglioma and one had an adrenal plus an extra-adrenal tumor. For all subsequent analyses, the latter patient was assigned to the extra-adrenal group. Surgery was curative in all cases, based on normal plasma metanephrines on the day of post-operative sampling [15]. Sampling took place from approximately one day to eight months before surgery, and approximately one month to three years after tumor removal. Post-operative samples were collected, on average, one year after pre-operative sampling. Samples were analyzed by means of proton NMR spectroscopy ~4.5 years and 3 years after collection (median values, for respectively pre- and post-operative samples).

**Table 1:** Patient and sample characteristics

Clinical factors/covariates	No of patients in each group/Average/middle value—[Range]
Sex (F/M) (36)	27/9
Age (years) (36)	51 [26–74]
Body Mass Index (kg/m2) (34)	25.5 [17.7–33.9]
Center of Origin (36)	18 Warsaw, 12 Dresden, 2 Nijmegen, 2 Munich, 1 Lubeck, 1 Würzburg
Catecholamine Phenotype (Adrenergic/Nonadrenergic) (36)	18/18
Tumor Location (36)	31 adrenal (1 bilateral), 4 extra-adrenal, 1 adrenal + extra-adrenal
Tumor Size (maximum diameter, cm) (36)	4.8 [2–16]^a^
PPGL-related gene mutation (36)	22 sporadic, 3 NF1, 4 RET, 1 SDHB, 2 SDHC, 2 SDHD, 1 EPAS1, 1 VHL
Pre-operative plasma metanephrines (36)	
Metanephrine (pg/ml) Upper reference limit – 88 pg/ml	109 [7.2–2306.1]^a^
Normetanephrine (pg/ml) Upper reference limit – age-specific	1070 [40.8–8774.3]^a^
3-methoxytyramine (pg/ml) Upper reference limit – 17 pg/ml	22 [5.4–459.4]^a^
Total metanephrines (pg/ml)	1456 [168.3–9451.3]^a^
Post-operative plasma metanephrines (35)	
Metanephrine (pg/ml) Upper reference limit – 88 pg/ml	21 [0.7–76.3]^a^
Normetanephrine (pg/ml) Upper reference limit – age-specific	80 [28.4–195.6]^a^
3-methoxytyramine (pg/ml) Upper reference limit – 17 pg/ml	14 [1.8–30.8]^a^
Total metanephrines (pg/ml)	114 [44.8–256.8]^a^
Pre-operative 24 h urine catecholamines (34)	
Epinephrine (ug/24 h) Upper reference limit – 15 ug/24 h	27 [0.3–206.3]^a^
Norepinephrine (ug/24 h) Upper reference limit – 60 ug/24 h	126 [4.8–2775.9]^a^
Dopamine (ug/24 h) Upper reference limit – 382 ug/24 h	223 [27.2–5160.7]^a^
Total urine catecholamines (ug/24 h)	383 [35.8–8024.3]^a^
Post-operative 24 h urine catecholamines (23)	
Epinephrine (ug/24 h) Upper reference limit – 15 ug/24 h	2 [0.4–8.8]^a^
Norepinephrine (ug/24 h) Upper reference limit – 60 ug/24 h	20 [7.3–42.2]^a^
Dopamine (ug/24 h) Upper reference limit – 382 ug/24 h	183 [100.0–324.7]
Total urine catecholamines (ug/24 h)	208 [116.5–351.8]
Pre-operative morbidity	
Hypertension (yes/no/unknown)	31/4/1
Diabetes mellitus (yes/no/unknown)	8/26/2
Post-operative morbidity	
Hypertension (yes/no/unknown)	12/12/12
Diabetes mellitus (yes/no/unknown)	3/28/5
Sampling	
Time between pre- and post-operative sampling (days)	341 [34–1159]^a^
Pre-operative sample age (days)	1629 [732–2841]
Time between pre-operative sampling and surgery (days) (30)	24 [1–252]^a^
Post-operative sample age (days)	1252 [318–2023]
Time between post-operative sampling and surgery (days) (30)	366 [17–1112]^a^

^a^Factors for which non-normality was proven and median is reported instead of mean

in parentheses: number of patients for which the information was known per factor

### Baseline metabolomics

**Fig. 1:**
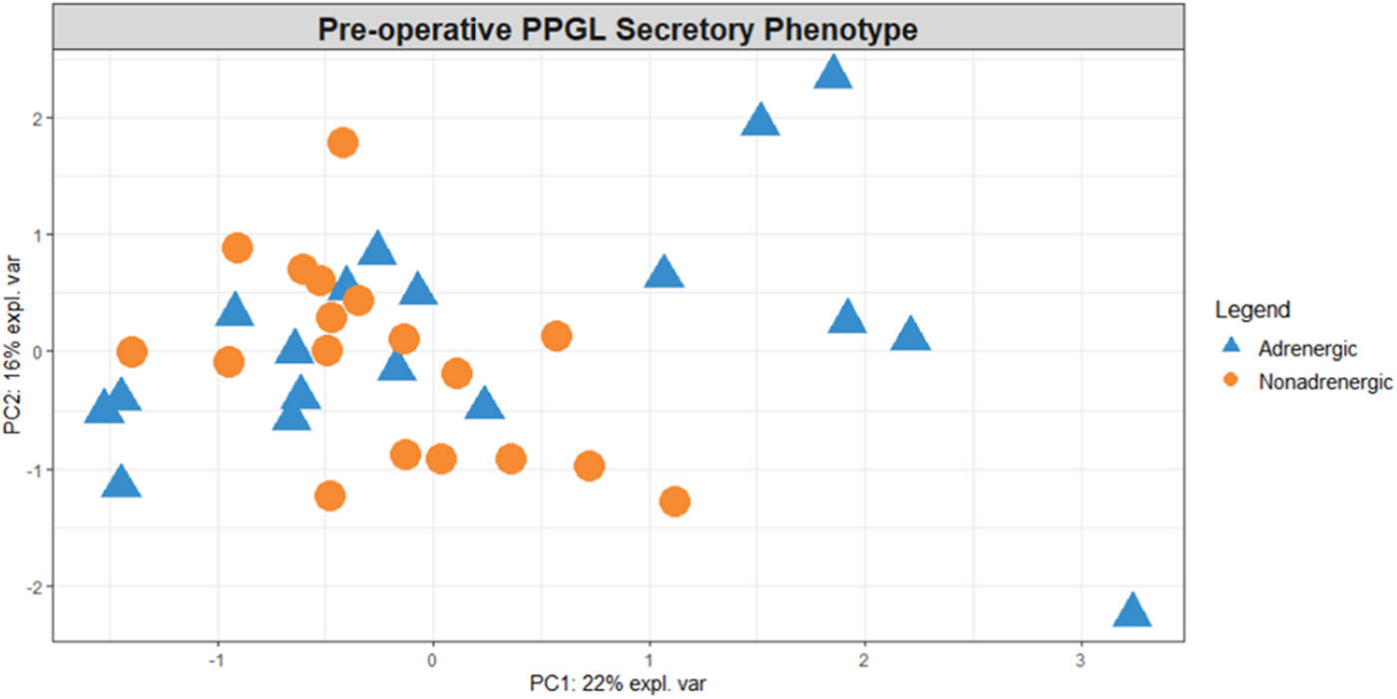
Pre-operative PCA score plot explaining 38% of the variance. Samples are identified based on color and shape, with patients with an adrenergic tumor represented by blue triangles and patients with a nonadrenergic tumor by orange dots

Processing of the plasma NMR spectra resulted in 91 peaks, corresponding to a total of 30 metabolites. Five peaks could not be assigned to known metabolites, and were listed as “unknowns”. Collected data were determined to have significantly higher amount of biological variance compared to technical variance. The influence of analytical factors was investigated and was not found to affect results on a multivariate level. Even so, we found one significant correlation between the serine peak at 3.939 ppm and the order pre-operative samples were analyzed within batches (*p* = 7e–03, rho = 0.61). The underlying cause of this correlation remains unclear. Additional information on data quality can be found in the supplementary information of this paper, section 3.2 and Fig. 1S.

The investigation of the relationship between individual plasma metabolites and clinical or preanalytical factors (indicated in section 3.3) revealed tyrosine (peak at 7.18 ppm) as significantly higher in patients with a high BMI, as compared to those with a low BMI (cutoff: 25 kg/m^2^, *p* = 0.02). No other individual metabolites were significantly different between groups defined by the clinical or preanalytical factors (data not shown). Multivariate regression analysis was used to investigate the interaction between all factors and the metabolome. No significant multivariate regression models were found, indicating that the clinical and technical factors considered were not found to have a significant overall effect on the pre-operative patient metabolome (Table 3S).

No differences between groups of patients defined by clinical or preanalytical factors were found using multivariate statistics at baseline, except between catecholamine phenotypes (classification accuracy 72%, *p* = 0.001; Table 1S). This separation can be observed in the PCA score plot presented in Fig. 1. The list of important metabolites for this separation, based on PLSDA (Table 2S), was comprised of higher levels of ketone bodies (acetoacetate and 3-hydroxybutyrate), creatine, pyruvate and serine in patients with nonadrenergic than in patients with adrenergic tumors. Glycerol, acetylcarnitine, dimethyl sulfone, succinate, lactate and creatinine, as well as an unknown metabolite with a peak at 3.26 ppm, were higher in patients with adrenergic than in patients with nonadrenergic tumors. We found no significant results comparing mutation clusters, possibly because of sample size. In addition, we did not find any significant results distinguishing groups of pre-operative samples defined by using the median number of days before surgery as a cutoff, using multivariate (*p* = 0.925, Table 1S, supplementary information), or univariate (data not shown) statistics. Spearman correlation tests did not reach significance either (data not shown). We applied the same univariate tests on post-operative sample data, but again found no significant results (data not shown).

**Table 2:** Relevant metabolites for separating pre- from post-operative samples

METABOLITE	NMR PEAK (ppm)	PRE-OP	MEDIAN FOLD CHANGE (PRE/POST)^a^	MAD FOLD CHANGE (PRE/POST)^a^	Subgroup analysis: *Female patients*	Subgroup analysis: *BMI* *<* *25* *kg/m*^*2*^	Subgroup analysis: *days between pre- and post-operative sampling* *>* *=median*
3-hydroxybutyrate^b^	2.313	↑	1.139	0.571	S		
	2.370	↑	1.686	1.139	S		S
3-hydroxybutyrate/Proline^b^	4.133	↑	1.170	0.339	S		S
Acetoacetate	2.262	↑	1.136	0.650	S		S
Dimethyl sulfone^b^	3.137	↓	0.939	0.407	S		S
Glucose^b^	5.220	↑	1.192	0.238	S, s	s	S,s
	5.227	↑	1.184	0.216	S, s	s	S,s
Glycine^c^	3.548	↑	1.031	0.203	S		S
Histidine/Phenylalanine^c^	3.126	↓	0.823	0.223			S
Histidine/Phenylalanine/Serine^c^	3.985	↓	0.873	0.152			S
Lactate^b^	4.080	↓	0.980	0.278			S
	4.094	↓	0.909	0.255	S		
	4.108	↓	0.961	0.264	S		S
Lysine^b^	2.997	↓	0.959	0.226	S		
	3.013	↓	0.933	0.218	S		
Methanol^c^	3.346	↑	1.132	0.332	S		S
Ornithine^b^	3.041	↓	0.918	0.251	S		S
Proline^b^	1.996	↑	1.152	0.222			S
	2.060	↑	1.129	0.311			S
	3.312	↑	1.249	0.276			S
Pyruvate^c^	2.356	↑	1.086	0.326	S		S
Succinate/3-hydroxybutyrate^b^	2.389	↑	1.099	0.364	S		
Tyrosine^c^	6.892	↓	0.868	0.320	S		
	7.185	↓	0.820	0.169		s	
Unknown metabolite(s)	3.162	↑	1.060	0.328			S
	3.284	↓	0.918	0.410			S
	3.670	↑	1.324	0.541		s	

S: Peaks found important using multivariate models

s: Peaks found significant with univariate statistics

^a^A median fold change above 1 signifies a metabolite which is higher in pre-, whereas below 1 is higher in post-operative samples. Median Absolute Deviation is the measure of spread of the median value of the fold change population, and can be used along with the median fold change to understand which metabolites alter their levels more, relative to other metabolites.

^b^Peak identity determined by visual inspection + 2D NMR along with experiments on filtered plasma at pH 2.5

^c^Peak identity determined by visual inspection + 2D NMR + spiking experiments

The trend of increase or decrease in pre-operative samples, was determined based on paired fold changes generated based on the subgroup data used for univariate statistics

Due to the nature of untargeted NMR, several metabolites can have more than one peak assigned to their name, and due to signals arising from multiple metabolites contributing to several single peaks, multiple names are listed for several entries

### Pre- vs. post-operative metabolomics

**Fig. 2:**
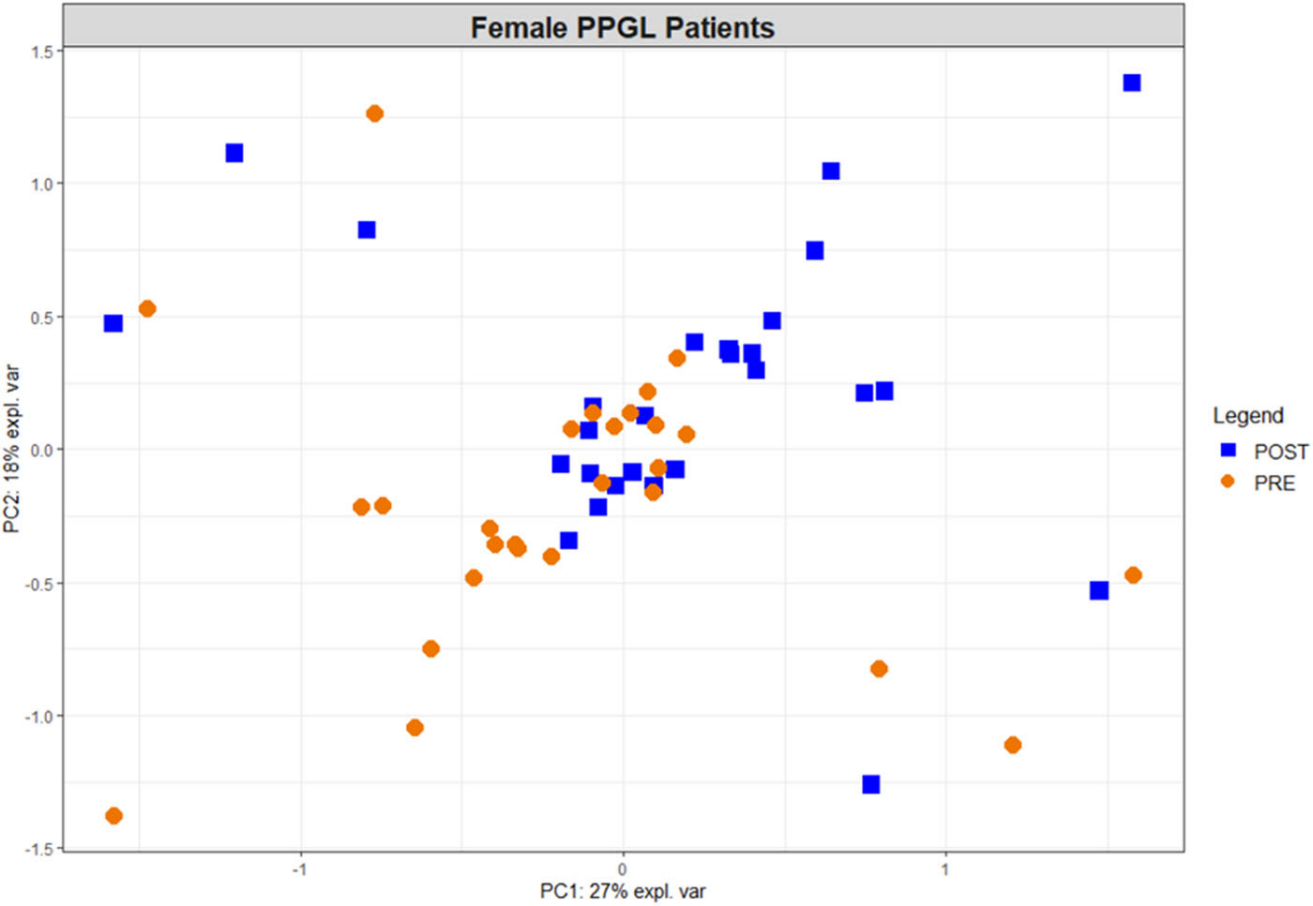
Paired Pre- vs. Post-operative PCA plot of female patients explaining 45% of the total variation present in the dataset. Multilevel analysis subtracts variation related to patient individuality by subtracting the mean of the two measurements per patient from each measurement, essentially resulting in a paired PCA model. As a result, each sample’s counterpart can be found on the opposite side of the center of the plot. Pre-operative samples samples are presented as orange dots whereas post-operative samples are blue squares

We evaluated possible differences between the pre- and post-operative metabolomes in PPGL patients with a paired PCA analysis (supplementary information, section 2.3). In this unsupervised analysis, i.e., without using the sample group (pre/post) information, we observed a tendency for separation between pre- and post-operative samples collected from females (Fig. 2). We subsequently proceeded with supervised statistical analyses on the complete dataset as well as subgroups, (paired PLSDA and paired univariate tests) to extract the metabolic signature of this separation.

The multilevel PLSDA model separating the complete patient set of pre- from post-operative samples was not found to be significant (*p* = 0.091, table 4S). Subgroup analyses resulted in a significant model for the female patient set (*p* = 0.044, table 4S). The metabolites acetoacetate, glycine, 3-hydroxybutyrate, glucose, pyruvate, methanol and succinate were increased in pre-operative samples (Table 2). On the other hand, ornithine, tyrosine, lactate, dimethyl sulfone and lysine were increased in post-operative samples. Another significant subgroup model was for patients post-operatively sampled more than the median of 341 days after pre-operative sampling. This model (days between pre- and post-operative sampling *>* =median, *p* = 0.008) added proline and an unknown NMR peak at 3.16 ppm (increased preoperatively), as well as histidine/phenylalanine and an unknown NMR peak at 3.28 ppm (decreased preoperatively) to the overall signature. In the subgroup of patients with a low BMI (*<*25 kg/m^2^), tyrosine was found to be significantly decreased pre-operatively (*p* = 0.009), and an unknown metabolite with a peak at 3.67 ppm was found to be significantly increased pre-operatively (*p* = 0.019).

**Fig. 3:**
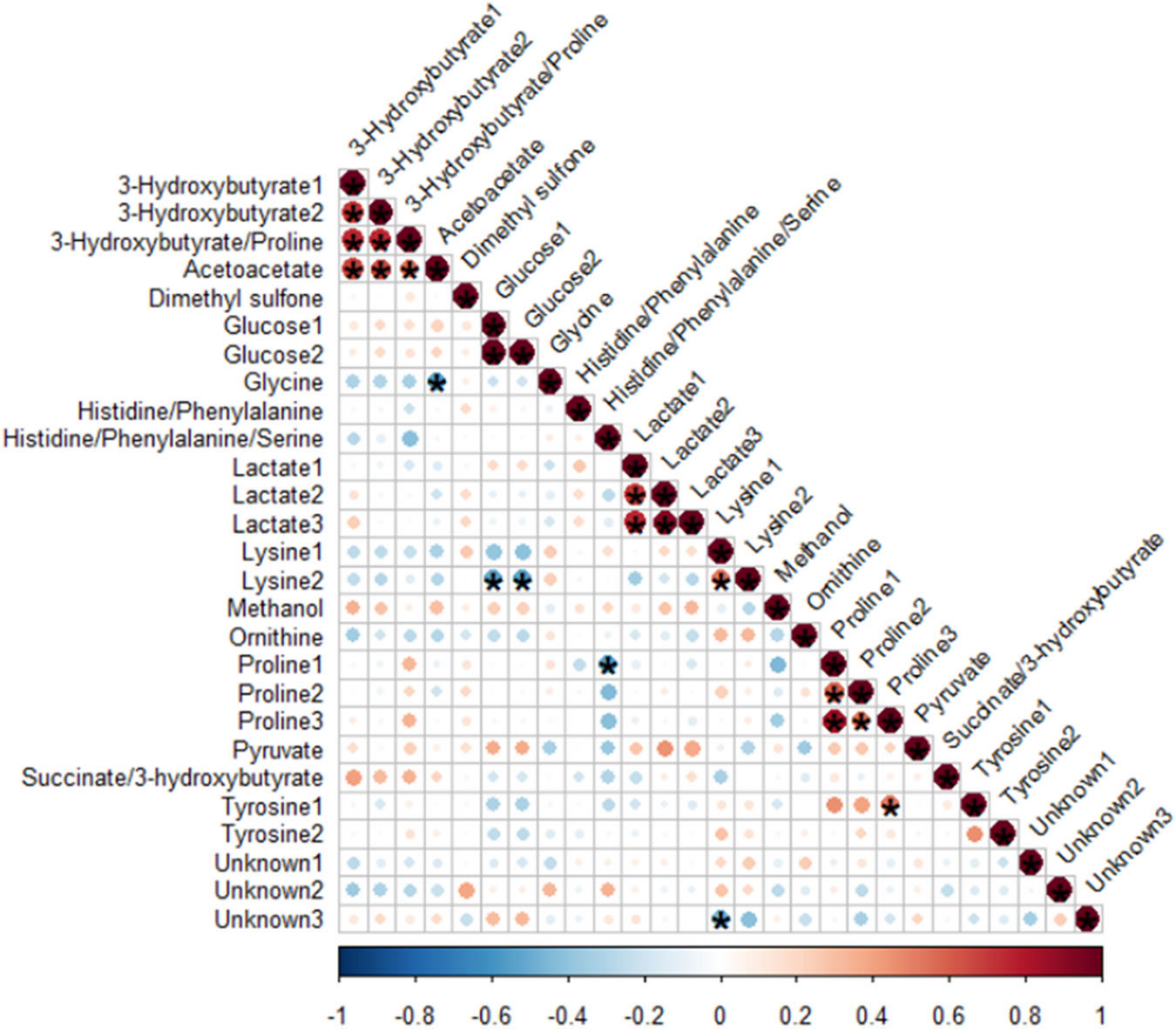
Correlation plot associating each important peak delta with every other. Metabolite delta was determined by subtracting each metabolite’s post-operative from its pre-operative levels. The estimate of the Spearman correlation coefficient (rho) determines both the color and size of each dot. Large and red dots represent strong correlations, small dots are weak correlations and blue dots are negative correlations. The associated significance for each correlation is depicted as an asterisk (*)

The differences between important metabolite levels before and after surgery (delta) were subsequently investigated for correlations with clinical factors. Starting with multivariate regression, the analysis resulted in no significant models (table 5S). In terms of univariate analyses, Spearman correlations between clinical factors and important metabolites can be found in Fig. 3. Significant positive correlations were found between acetoacetate and 3-hydroxybutyrate (*p* = 6e–03, rho = 0.56, *p* = 2e–04, rho = 0.68, *p* = 2e–04, rho = 0.67).

Glucose delta correlated positively and significantly with total levels of plasma metanephrines (*p* = 0.010, rho = 0.54). No significant correlations were found between important metabolites and urine catecholamine outputs. In regards to correlations between metabolites, glucose correlated significantly and negatively with lysine (*p* = 0.012), rho = −0.53, (*p* = 0.010, rho = −0.54). Acetoacetate correlated negatively with glycine(*p* = 7e–03, rho = −0.56), indicating stable levels of one metabolite coinciding with alterations of the other, since they were both increased before surgery. Similarly, the positive significant correlation (*p* = 0.015, rho = 0.52) between proline and tyrosine shows that the levels of one metabolite correlate negatively with the levels of the other, since proline was increased pre-operatively, while tyrosine was decreased. Proline also had a negative significant correlation(*p* = 9e–03, rho = −0.55) with histidine/phenylalanine/serine (convoluted peak with signal overlap). Other correlations can be observed in Figs. 3 and 2S.

## Discussion

The variability with which PPGLs can present clinically, along with their widespread extent of sympathetic activation [5], render their study an appropriate research topic for metabolomics. In the present paper, we report the metabolic alterations we found in patients with PPGL, using untargeted NMR metabolomics of plasma samples taken both before and after surgical removal of the tumor. Specifically, metabolites related to glucose metabolism were found to be affected, along with compounds linked to muscle wasting in literature.

In the previous study by Erlic et al. [13] on the impact of PPGL on plasma metabolome, a targeted approach was taken using LC-MS metabolomics. Even though untargeted NMR detected a different set of analytes (with some overlap), our results partially agree in terms of a signature of PPGL metabolic impact. Furthermore, we found metabolites with altered levels before compared to after surgery which had not been reported in relation to PPGL.

At baseline, we found differences based on catecholamine biochemical phenotype that coincide with expected effects of adrenergic vs. nonadrenergic tumors on patient metabolism. Specifically, glycerol, which is a product of lipolysis, a process enhanced by epinephrine more than by norepinephrine [25], was higher in patients with adrenergic compared to those with nonadrenergic tumors. Conversely, ketone bodies, indicating enhanced ketogenesis, which has been shown to be stimulated by norepinephrine at pathophysiological concentrations [26], were higher in patients with nonadrenergic compared to those with adrenergic tumors. In the targeted study by Erlic et al. [13], the main differences at baseline related to gender, rather than catecholamine biochemical phenotype. These differences with our study could be due to our small number of male patients, along with their not measuring glycerol or ketone bodies, which were the most important separators at baseline. Another finding was significantly higher tyrosine levels in patients with high compared to low pre-operative BMI, a result supported by targeted metabolomics [13]. Previous work on PPGL has proved that SDHx-PGLs rely on the Warburg effect [27]. However, we could not find differences between SDH and non-SDH patients within our cohort, probably because of the differences in studying plasma compared to intracellular environments.

Paired comparisons of baseline samples from their respective post-operative controls resulted in two subgroup significant separations, which yielded an overall signature composed of, among others, glucose metabolism-related compounds pyruvate, lactate, histidine and glucose itself. In line with the targeted metabolomics study by Erlic et al. [13], where a decrease of a hexose was found after surgery, we found significantly increased glucose levels pre-operatively as expected, given the known PPGL effects on glucose homeostasis [6]. Histidine peaks had decreased intensity pre-operatively on average, as in the Erlic et al. study [13], with the authors concluding a relationship with diabetes. However, a technical difficulty regarding histidine is that its NMR peaks did not correlate with each other due to overlap with signals from other metabolites, and they were only found to be important pre- vs. postoperative discriminators in patients post-operatively sampled more than the median of 341 days after pre-operative sampling. Although catecholamines have been shown to cause hyperlactatemia [28], the decreased pre-operative lactate may be explained by decreased glycolysis, which is in line with a study by Wu et al. [29]. Essentially, in the presence of PPGL, the equilibrium of the Cori cycle appears to be shifted toward the direction of increased gluconeogenesis, as evidenced by increased pyruvate, and decreased glucose consumption by tissue glycolysis [28], as lactate levels drop pre-operatively. Based on the positive correlation between pyruvate and lactate, it would seem that the mechanisms leading to high glucose operate competitively. However, the exact mechanism for these phenomena remains unclear. Another finding of our study was increased ketone bodies before compared to after surgery in most patients (23/36) and with a fold change of 1.14 for acetoacetate in females (Table 2). Also, the correlation of glucose with the ketogenic amino acids tyrosine and lysine, may indicate decreased insulin secretion [30]. Although ketoacidosis has only been shown in a handful of PPGL cases [31], ketogenesis can be aroused by a switch from glycolysis to fatty acid metabolism in tissues, a result of either insulin resistance or decreased insulin secretion [32], both of which have been associated with stimulation of adrenergic receptors [6]. The relationship between glycine and ketogenesis, evidenced by its significant correlation to acetoacetate, may be based on the amino acid’s ability to enhance insulin secretion [33].

BMI can be slightly lower in patients with a PPGL [6]. In our study, we were unable to confirm this due to limited post-operative information on BMI (only known for 22/36 patients). However, our results appear to partially agree with the study by Cala et al. [34], which described the metabolomic profile of cachexia in patients with other types of tumors, with lysine, ornithine, histidine and tyrosine being decreased in cachectic patients. Tumor-related cachexia is postulated to be related to gluconeogenesis [35], which results in withdrawal of proteins and lipids from non-tumor tissue for energy purposes. It’s conceivable, based on these findings, that PPGL-mediated muscle atrophy is intertwined with the changes in glucose homeostasis. Although cachexia is not clinically evident in patients with PPGL, skeletal muscle mass is decreased in patients with PPGL [36]. The pre-operative decrease in ornithine is further supported by the targeted metabolomics study [13]. Our hypotheses on the pathways impacted by the presence of a PPGL seem to be corroborated by both our results and literature, but to prove and understand them further research is needed.

Several metabolites not previously considered as markers of PPGL appeared to play a potential role in PPGL patient metabolism. Specifically, proline, dimethyl sulfone and methanol potentially contribute to PPGL metabolic impact. In addition, ketogenesis has not been demonstrated to be enhanced due to PPGL often, and we have no explanation for the overall pre-operative increase in succinate (which overlaps with 3-hydroxybutyrate, Table 2).

A limitation of the study was the small sample size for subgroup analysis. For example, males were underrepresented. It is possible that certain comparisons were affected by this limited statistical power. It would be interesting to investigate separately e.g., the effects of nonadrenergic tumors on patient metabolome (pre vs. post), and compare the results to those from an adrenergic tumor investigation, especially since this was a significant difference for patients at baseline. It would also be interesting to investigate metabolic differences between samples collected from patients belonging to different mutation clusters. Also, not all metadata information was complete, especially after surgery, for factors that may have a significant impact on patient metabolome. For example, medication, as well as the time of weight measurements could be important but were not taken into account, and neither was pre vs. post-operative patient and sample age differences, which were unavoidable as patients were followed up after surgery. This limited information was investigated directly on recorded NMR spectra, but due to our data processing approach (supplementary information, section 2.2) peaks unique in a few samples were not retained in the final dataset. Another characteristic not considered was sample hemolysis level. Though patients adhered to an overnight fast, detailed dietary information, which could influence ketone body levels, was unavailable. Finally, untargeted NMR metabolomics is hindered by peaks not assigned to known metabolites, even after specialized experiments were performed, as well as signal overlap, which resulted in mixed identities for several peaks, and a low correlation between the important tyrosine peaks.

In conclusion, the comparison of pre- to post-operative samples led to the discovery of differences related to glucose metabolism, in particular increased ketogenesis and gluconeogenesis. In addition, metabolites previously linked to muscle wasting were found to be decreased pre-operatively. Before surgery, patients with nonadrenergic tumors were characterized by alterations in metabolite levels fitting with decreased lipolysis, as well as increased ketogenesis, compared to patients with adrenergic tumors. Overall, our findings corroborate previous conclusions about the effects of PPGL on glucose homeostasis and body mass, and offer possible explanations as to the biochemical mechanism underlying these effects.

## Data Availability

Data used for the purposes of this work will be provided upon request by the corresponding author to any interested readers.

## Supplementary infomation

Supplementary information
