# Supplementary material for: Pre- versus post-operative untargeted plasma nuclear magnetic resonance spectroscopy metabolomics of pheochromocytoma and paraganglioma
Source: Endocrine. 2021 Sep 18;75(1):254–65. doi: 10.1007/s12020-021-02858-z (PMC8763816; doi:10.1007/s12020-021-02858-z)
Supplement: Supplementary file 1 — Supplementary information [file 12020_2021_2858_MOESM1_ESM.docx]

# Pre- versus Post-Operative Untargeted Plasma Nuclear Magnetic Resonance Spectroscopy Metabolomics of Pheochromocytoma and Paraganglioma

Journal name: Endocrine

Nikolaos G Bliziotis1*, Leo AJ Kluijtmans1, Sebastian Soto1, Gerjen H Tinnevelt2,

Katharina Langton3, Mercedes Robledo4, Christina Pamporaki3, Udo FH Engelke1,

Zoran Erlic5, Jasper Engel6, Timo Deutschbein7,8, Svenja Nölting9, Aleksander Prejbisz10,

Susan Richter11, Cornelia Prehn12, Jerzy Adamski12,13,14,15, Andrei Januszewicz10,

Martin Reincke9, Martin Fassnacht7,16,17, Graeme Eisenhofer3,11, Felix Beuschlein5,9,

Matthias Kroiss7,9,16,17, Ron A Wevers1, Jeroen J Jansen2, Jaap Deinum18

and Henri JLM Timmers18*

1Department of Laboratory Medicine, Translational Metabolic Laboratory, Radboud University Medical Center, Nijmegen, the Netherlands, 2Department of Analytical Chemistry, Institute for Molecules and Materials, Radboud University, the Netherlands, 3Department of Medicine III, University Hospital Carl Gustav Carus, Technische Universita¨t Dresden, Dresden, Germany, 4Hereditary Endocrine Cancer Group, Spanish National Cancer Research Centre (CNIO), Madrid, Spain and Centro de Investigacio´ n Biome´dica en Red de Enfermedades Raras (CIBERER), Madrid, Spain, 5Klinik fu¨ r Endokrinologie, Diabetologie und Klinische Erna¨hrung, Universita¨tsspital Zu¨ rich, Switzerland, 6Biometris, Wageningen UR, Wageningen, The Netherlands, 7Schwerpunkt Endokrinologie/Diabetologie, Medizinische Klinik und Poliklinik I, Universita¨tsklinikum Wu¨ rzburg, Germany, ^8^Medicover Oldenburg MVZ, Oldenburg, Germany, 9Medizinische Klinik und Poliklinik IV, Klinikum der Universita¨t,Ludwig-Maximilians-Universität, Mu¨ nchen, Germany, 10Department of Hypertension, Institute of Cardiology, Warsaw, Poland, 11Institut fu¨ r Klinische Chemie und Labormedizin, Universita¨tsklinikum Carl Gustav Carus, Dresden, Germany, 12Helmholtz Zentrum Mu¨ nchen, Research Unit Molecular Endocrinology and Metabolism, Neuherberg, Germany, 13Institute of Biochemistry, Faculty of Medicine, University of Ljubljana, Ljubljana, Slovenia, 14Institute of Experimental Genetics, Helmholtz Zentrum München, German Research Center for Environmental Health, Neuherberg, Germany, 15Department of Biochemistry, Yong Loo Lin School of Medicine, National University of Singapore, Singapore, Singapore, 16Core Unit Clinical Mass Spectrometry, University Hospital Wu¨ rzburg, Wu¨ rzburg, Germany, 17Comprehensive Cancer Center Mainfranken, Universita¨t Wu¨ rzburg, Wu¨ rzburg, Germany, 18Department of Internal Medicine, Radboud University Medical Center, Nijmegen, the Netherlands.

*: Corresponding authors. ORCID IDs/emails:

0000-0001-7775-6613/nick.bliziotis@radboudumc.nl (Nikolaos G Bliziotis)

0000-0002-1208-5748/henri.timmers@radboudumc.nl (Henri JLM Timmers)

## Supplementary Information

### 2 Materials and Methods

#### 2.2 Untargeted NMR metabolomics

As stated in the manuscript, the method developed in our previous work was used with some modifications^1^. A maximum of 15 samples per batch to limit intra-batch variability was selected, based on robust Principal Component Analysis (PCA)^2^ results (R package "rospca"^3^), which showed QC samples outlying after 18 NMR experiments. Samples were centrifuged at room temperature for the first three batches and at 4°C for all the rest. A Bruker DRX AVANCE spectrometer equipped with a triple resonance inverse 5 mm probe head operating at 500.13 MHz was employed for analyzing samples. Both the sequence and batch number of all samples were recorded for estimating technical variability. Longitudinal Eddy-Current Delay (LED) spectra were recorded and processed as previously described^1^. Spectra with high line width were not used for further analysis (>1.2 Hz). Areas corresponding to macromolecules, water, glucose and noise were excluded from data processing (areas above 10, 5.35 to 5.24, 5.15 to 4.40, 3.91 to 3.68, 3.54 to 3.36, 3.26 to 3.19, 1.30 to 1.10, 0.90 to 0.75, and below 0 ppm). Peaks at 3.185, 2.21 and 1.904 ppm were also excluded because of their strong correlation with the run order of samples. Even so, both QC samples and HV samples were separated based on run order and batch number, although the same was not found for pre- or post-operative samples that were analyzed for the purposes of this study. R studio version 1.1.463^4^ running R version 3.4.4^5^ was used for loading the R package “SPEAQ”^6^ for obtaining the peak table. Due to their high number, SPEAQ was not possible to perform on all samples simultaneously. Hence, a script for aligning different SPEAQ batches was developed. R version 3.6.3 was used for all subsequent steps. After this batch alignment step, the ethanol peaks at 3.65 ppm, the highly variable histidine peaks at 7.06 and 7.81 ppm, the macromolecule peak at 2.02 ppm, the peaks at 2.21, 3.185 and 1.904 ppm, which were found to correlate with the order of HV sample analysis, were all removed, along with peaks not present in at least 80% of samples belonging to either the QC or HV group^7^. Probabilistic Quotient Normalization (PQN^8^) was applied as a normalization method, using as reference the median spectrum ignoring non-detects of a set of samples that were drawn from 98 healthy volunteers at the University Hospital Dresden for the PMT study. Next, 17 peaks with a coefficient of variation of more than 30% in QC samples were removed. And finally, after missing value estimation with the k-nearest neighbors (k-NN) method^9^ (k=10), the generalized log transformation (GLOG^10^) based on 133 Quality control (QC) samples was applied using R package “LMGene”^11^. The QC samples were prepared by pooling plasma obtained from 390 anonymized plasma samples to a total volume of about 450 mL, which was subsequently aliquoted into 1 mL batches and stored at -80°C until analysis. As a normalization and GLOG transformation optimization step, the process was repeated after the HV and QC sample groups were relieved of outliers detected by means of robust PCA^2^. The resulting dataset was directly used for multivariate statistics, whereas for univariate statistics the resulting dataset before missing value estimation and after applying CV filtering, was employed^12^. QC and HV samples were separated according to run order and batch analyzed, but this effect was not observed for any of the groups under investigation, so it was not considered to bias our results. All parameters and scripts can be found on the first author’s GitHub page (https://github.com/NickBliz/PPGL-PRE-VS-POST) and data can be provided upon request.

To aid in peak assignment, additional methods were employed. Specifically, 2D NMR, namely correlation spectroscopy (COSY, cosyprqf) and J-resolved (JRES, jresgpprqf) pulse sequences allowed for investigating correlations between peaks and peak multiplicity, respectively. These experiments, along with 1D NMR on filtered samples in pH 2.5^13^, allowed for a number of peak identities to be confirmed, and some unknowns to be assigned to metabolites. Finally, QC samples were spiked with a number of metabolites to confirm assignments (Tables 2, 2S).

#### 2.3 Data Analysis and Statistics

Multivariate Analysis (MVA) was performed on mean-centered data by employing the MixOmics R package^14^. We used PCA^15^ to discover trends; Partial Least Squares Discriminant Analysis (PLSDA)^16^ was used both for investigating the metabolic signature of the differences between pre- and post-operative samples as well as for investigating differences between patients at baseline according to a clinical factor. Metabolomics data for PLSDA were not scaled, whereas patient metadata for PLS regression were scaled. The number of latent variables of each PLSDA model as well as its quality, was chosen/assessed by double cross validation (CV2)^17,18^, by leaving out either one sample for non-paired, or a pair of samples originating from the same patient for paired (multilevel) models and predicting their labels based on the model and using the Mahalanobis distance. The maximum number of latent variables for PLSDA models was 10, and for each inner cross validation loop the minimum number of components with the maximum accuracy was chosen, based on predictions using the Mahalanobis distance. PLSDA model significance was determined based on the result of n=1000 permutation tests^17,18^, in which the Y value of a random number of samples were swapped for those of an equal number of samples in the dataset and vice-versa. Important variables were determined based on their Variable Importance in the Projection (VIP) score^19^ (median outer-loop VIP score above 1 after double cross validation, or CV2) in PLSDA models. In addition, classic Partial Least Squares (PLS)^16^ was used as a multivariate regression (MVR) method, to investigate the influence of clinical factors on the patients' metabolome. Again, double cross-validation was used for optimization and evaluation of the model. Clinical factors investigated can be found in Table 1 and include pre- values which were more complete than their respective post-operative counterparts. Quality of PLS models was determined based on Q2 and *p-*values obtained from double cross validation and permutation testing respectively. When analyzing the metabolomic differences between pre- and post-operative patient samples, the Multilevel (ML) approach^20^ was used for the paired multivariate models. The Multilevel approach subtracts the mean of each patient's samples to highlight the difference between pre- and post-operation, by specifying the study design to the MixOmics R command. Thus, for pre- vs. post-operative sample analyses, we employed paired PCA and PLSDA.

Permutation testing was repeated 1000 times for each model investigated, by enabling multi-core parallel processing^21^ on a supercomputer^22^ for R, version 3.6.2. The original model’s significance was measured by computing the *p*-value compared to the permuted models, using the balanced accuracy (average of sensitivity and specificity) for PLSDA, number of misclassifications for ML-PLSDA, or Q2 of all latent variables for PLS (a.k.a. total Q2) and the formula in the paper by Szymanska et al^17^. The balanced accuracy is the unbiased version of the classic classification accuracy, which takes into account relevant group size^23^. CV2 and permutation testing were carried out based on in-house developed R scripts. Instead of the classic threshold of 0.0975 for Q2^24^, we used the maximum value to select latent variables in each CV1 round, as the classic threshold was never exceeded.

Univariate statistical analysis methods were used to provide complementary results to multivariate tests^25^, and were performed using the "stats" R package^5^, to check for data normality^26^ and to discover significant differences between variables. For this purpose, data were not GLOG transformed. Univariate tests included the paired Wilcoxon test^27^ to discover any significant differences, at a significance level of 5%. The Spearman correlation^28^ estimate was used for investigating univariate non-parametric correlations between various factors (technical, clinical and biological) and peak intensities. Spearman's rho was deemed more appropriate than Kendall's tau for our purposes^29^, as the rho was also used in a similar study^30^ and could readily be compared to our results. The *p* values generated from the tests on the data collected, a false discovery rate correction^31^ was used to account for multiple testing, using the "p.adjust" command of the "stats" R package^32^. A corrected *p* value of less than 0.05 was accepted as statistically significant. Where appropriate, fold change was calculated either by dividing the median of the end response by the initial response samples (unpaired), or by averaging the quotient of the division of all end response samples over initial response samples (paired, division by patient). For building the correlation plot (Figure 2), the R package corrplot was employed^33^, along with the code provided by^34^.

### Results

#### 3.2 At baseline metabolomics

PCA of the metabolomics data for all patients (including pre- and post-operative samples), healthy volunteers and QC samples is shown in Figure 1S, with each sample analyzed having a unique metabolic signature that results in a single point in the plot. The first dimension explains 66% of the dataset's variation (Figure 1S), indicating how different the QC samples are from the study samples. The QC samples cluster closely together, whereas HV samples cluster the most out of all biological samples. Out of 111 peaks originally detected in the whole dataset and found in at least 80% of QC and HV samples, 3 were found to correlate significantly with the order HV samples were analyzed. These 3 peaks were subsequently excluded before data processing. Both HV samples could be separated based on the batch in which they were analyzed, and QC samples based on their run order within batches. However, neither batch or run order were found to influence results, based on low PLSDA classification accuracies for these factors on the datasets investigated. Even so, the Spearman/Wilcoxon tests found one significant peak correlating with the order preoperative samples were analyzed within batches/with different levels between samples analyzed at the beginning vs. the end of batches: the serine peak at 3.939 ppm (p=7e-03, rho=0.61/p=5e-03, fold change=1.377). The underlying cause of this correlation remains unclear. From the remaining 108 peaks, 17 were found to have a high coefficient of variation in the QCs (>30%), and so were left out before the analyses. The coefficients of variation of all 91 peaks in QC samples (which describe technical variation) were multiplied by two to be comparable to those of HVs, pre-operative PPGLs, and post-operative samples (which describe technical + biological variation) and were found to be lower in QCs than in any of the other groups of samples, with a paired t-test yielding significant results for the comparison of pre-operative peak CVs (*p*=1e-04), as well as post-operative (*p*=4e-03) and the combination of pre+post-operative (*p*=1e-05), but not for HVs (*p*=0.1). The median coefficient of variation in QC samples was found to be 0.102 in the final dataset, with a median absolute deviation of 0.070.


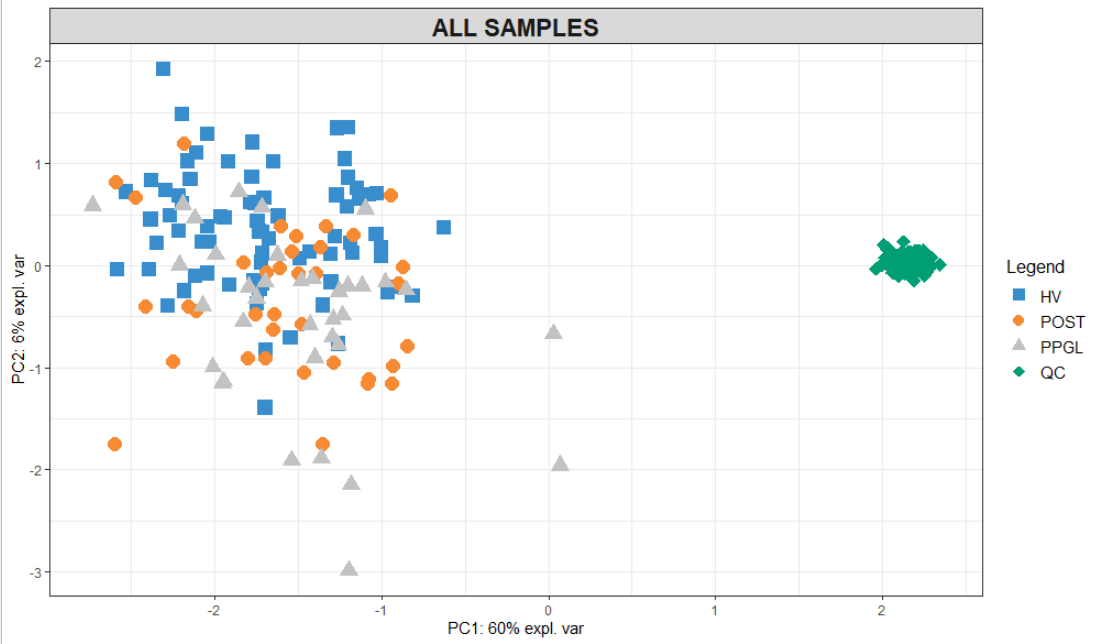


Figure 1S: PCA score plot of the complete dataset. This plot includes Healthy Volunteers (HV), Quality Controls (QC), Post-operative samples (POST) as well as Pre-operative samples originating from patients with PPGL (PPGL, pre-operative samples). Principal components 1 and 2 were used for the plot. QC samples were technical replicates and were aliquoted from pooled plasma collected from patients not included in the present study. The spread of QC samples can be indicative of technical variation associated to the data, which is significantly lower than biological.

Table 1S: The results from the Partial Least Squares Discriminant Analysis across all patients at baseline classified based on clinical factors. The model separating patients based on secretory phenotype was found to be significant (*p*<0.05, marked with an asterisk,*).

| Factor | p-value |
| --- | --- |
| center (Dresden/Warsaw) | 0.233 |
| sample age | 0.072 |
| Days before surgery | 0.925 |
| sex (male/female) | 0.057 |
| Patient age (45 yr) | 0.686 |
| BMI (25 *kg/m^2^*) | 0.655 |
| hypertension (y/n) | 0.446 |
| diabetes mellitus (y/n) | 0.539 |
| tumor location (adrenal/extra-adrenal) | 0.734 |
| **secretory phenotype (adrenergic/nonadrenergic)*** | **0.030** |
| Tumor size (5 cm) | 0.346 |
| total plasma metanephrines | 0.892 |
| total urine catecholamines | 0.523 |
| Batch | 0.714 |
| Run order | 0.320 |
| Cluster 1 vs. Cluster 2 | 0.529 |
| Presence vs. absence of mutation | 0.326 |
| Cluster 1 vs. all | 0.153 |
| Cluster 2 vs. all | 0.537 |
| SDH vs. SDH-negative | 0.159 |

Table 2S: The metabolic signature obtained from the significant patient secretory phenotype PLSDA model described in Table 1S. Each NMR peak was assigned to a metabolite, its levels determined higher or lower in patients with nonadrenergic than in those with adrenergic tumors (median values, based on the univariate non glog transformed data). The identity of each peak was either determined only by visual inspection and chemical shift values (no asterisk), visual inspection + 2D NMR and experiments on filtered plasma at pH 2.5 (single asterisk*), as well as visual inspection, 2D NMR + spiking experiments (double asterisks**). The final column indicates the importance in the model for each peak and is based on the median variable importance to the projection (VIP). Peaks were deemed important and are presented here if they had a median VIP of >1.

| METABOLITE | Peak Chemical Shift (ppm) | Nonadrenergic levels | Fold Change (N/A) | Importance in model |
| --- | --- | --- | --- | --- |
| Creatinine** | 4.041 | ↓ | 0.903 | 20 |
| Lactate* | 1.321 | ↓ | 0.937 | 19 |
| Serine/Phenylalanine/Histidine** | 3.973 | ↑ | 1.008 | 18 |
| Lactate* | 1.307 | ↓ | 0.947 | 17 |
| Lactate/Proline/3-Hydroxybutyrate* | 4.121 | ↓ | 0.960 | 16 |
| 3-Hydroxybutyrate/Proline* | 4.133 | ↑ | 1.045 | 15 |
| Threonine/Glycerol* | 3.567 | ↓ | 0.855 | 14 |
| Pyruvate** | 2.356 | ↑ | 1.290 | 13 |
| Unknown Metabolite | 3.262 | ↓ | 0.691 | 12 |
| Lactate* | 4.094 | ↓ | 0.989 | 11 |
| Lactate* | 4.108 | ↓ | 0.993 | 10 |
| Succinate/3-Hydroxybutyrate** | 2.389 | ↓ | 0.833 | 9 |
| Creatine** | 3.917 | ↑ | 1.084 | 8 |
| 3-Hydroxybutyrate* | 2.313 | ↑ | 1.204 | 7 |
| 3-Hydroxybutyrate* | 2.370 | ↑ | 1.117 | 6 |
| Creatine** | 3.021 | ↑ | 1.212 | 5 |
| Dimethyl sulfone* | 3.137 | ↓ | 0.848 | 4 |
| Acetylcarnitine** | 3.177 | ↓ | 0.779 | 3 |
| Acetoacetate | 2.262 | ↑ | 1.084 | 2 |
| Glycerol* | 3.555 | ↓ | 0.570 | 1 |

Table 3S: The results from four classic partial least squares models used as a regression method, based on data collected from samples at baseline. Each sample’s clinical values for each factor were used to describe their respective metabolomes. No significant models were found after CV2 and permutation testing (n=1000).

| Factors Considered | P VALUE |
| --- | --- |
| Total: sample age, sex, patient age, tumor size, plasma metanephrines, urine catecholamines, BMI, hypertension, diabetes, tumor location, secretory phenotype, days before surgery, center, run order, batch number | 0.590 |
| Technical: Sample age, days before surgery, center, run order, batch number | 0.348 |
| Biological: sex, patient age, tumor size, plasma metanephrines, urine catecholamines, BMI, hypertension, diabetes, tumor location, secretory phenotype | 0.982 |
| Clinical: tumor size, plasma metanephrines, urine catecholamines, BMI, hypertension, diabetes, tumor location, secretory phenotype | 0.957 |
| Excluding factors with missing information | |
| Total: sample age, sex, patient age, tumor size, plasma metanephrines, tumor location, secretory phenotype, center, run order, batch number | 0.331 |
| Technical: Sample age, center, run order, batch number | 0.255 |
| Biological: sex, patient age, tumor size, plasma metanephrines, tumor location, secretory phenotype | 0.460 |
| Clinical: tumor size, plasma metanephrines, tumor location, secretory phenotype | 0.892 |

#### 3.3 Pre- vs Post-operative Metabolomics

Table 4S: The results from multilevel (paired) PLSDA of Pre vs. Post samples originating from PPGL patients. Each row depicts the significance (p-value) of each model that was built upon each subset of the total (PVP) dataset. The only significant models (marked in an asterisk*) originated from the dataset including female patients and the dataset of patients that were post-operatively sampled a median of 341 days after pre-operative sampling. The final column is the number of important peaks for significant models that were summed in Table 2 to obtain the final signature of relevant metabolites. HT: Hypertension, DM: Diabetes mellitus.

| **Dataset** | **ML-PLSDA**  **P-value** | **Number of Important Peaks** |
| --- | --- | --- |
| PVP(n=72) | 0.091 |  |
| Samples from Dresden (n=24) | 0.579 |  |
| Samples from Warsaw (n=36) | 0.173 |  |
| BMI<25 *kg/m^2^* (n=30) | 0.217 |  |
| BMI>=25 *kg/m^2^* (n=38) | 0.664 |  |
| MALE(n=18) | 0.710 |  |
| **FEMALE(n=54)*** | **0.044** | **17** |
| AGE<45 yr. (n=26) | 0.645 |  |
| AGE>=45 yr. (n=46) | 0.391 |  |
| ADRENERGIC(n=36) | 0.127 |  |
| NONADRENERGIC(n=36) | 0.271 |  |
| ADRENAL(n=62) | 0.140 |  |
| TUMOR SIZE<5 CM (n=36) | 0.109 |  |
| TUMOR SIZE>=5 CM (n=36) | 0.702 |  |
| Days between pre- and post-operative sampling <median  (n=36) | 0.310 |  |
| **Days between pre- and post-operative sampling >=median (n=36)*** | **0.008** | **19** |
| HT Y PRE, N POST(n=18) | 0.310 |  |
| HT Y PRE, Y POST(n=24) | 0.128 |  |
| DM N PRE, N POST(n=52) | 0.232 |  |
| C1 (n=14) | 0.698 |  |
| C2 (n=14) | 0.521 |  |
| Presence of Mutation (n=28) | 0.751 |  |

Table 5S: The results from three classic partial least squares models used as a regression method, based on the differences in peak intensities between pre and post samples (delta). Each sample’s clinical values for each factor were used to describe their respective metabolomes. No significant models were found after permutation testing (n=1000).

| Factors Considered | P VALUE |
| --- | --- |
| Total: sex, patient age, tumor size, plasma metanephrines, urine catecholamines, BMI, hypertension, diabetes, tumor location, secretory phenotype, days between sampling, center | 0.092 |
| Biological: sex, patient age, tumor size, plasma metanephrines, urine catecholamines, BMI, hypertension, diabetes, tumor location, secretory phenotype | 0.100 |
| Clinical: tumor size, plasma metanephrines, urine catecholamines, BMI, hypertension, diabetes, tumor location, secretory phenotype | 0.062 |
| Excluding factors with missing information | |
| Total: sex, patient age, tumor size, plasma metanephrines, tumor location, secretory phenotype, days between sampling, center | 0.135 |
| Biological: sex, patient age, tumor size, plasma metanephrines, tumor location, secretory phenotype | 0.115 |
| Clinical: tumor size, plasma metanephrines, tumor location, secretory phenotype | 0.100 |


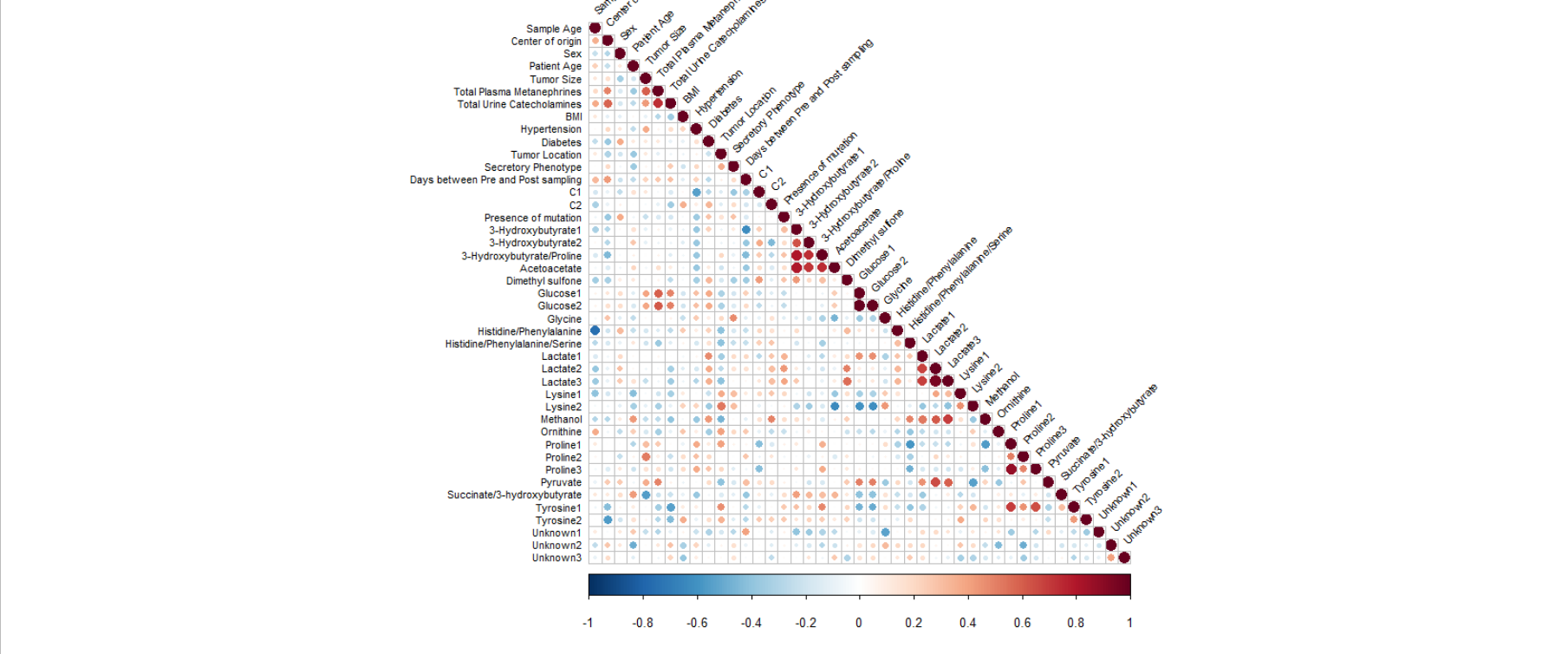


Figure 2S: Correlation plot associating each variable (factor or relevant metabolite) delta with every other. This plot is similar to Figure 3 of the result section, excluding the asterisks for marking significance but including all correlations with all clinical factors investigated. A total of 32 patients were used for this plot, as all factor information was available for these patients.

### References

1. Bliziotis NG, Engelke UFH, Aspers RLEG, et al. A comparison of high-throughput plasma NMR protocols for comparative untargeted metabolomics. *Metabolomics*. 2020;16(5):64. doi:10.1007/s11306-020-01686-y

2. Hubert M, Reynkens T, Schmitt E, Verdonck T. Sparse PCA for High-Dimensional Data With Outliers. *Technometrics*. 2016;58(4):424-434.

3. Tom Reynkens. rospca: Robust Sparse PCA using the ROSPCA Algorithm. 2018.

4. R studio team. RStudio: Integrated Development for R. RStudio, Inc., Boston, MA URL http://www.rstudio.com/. 2016.

5. R core team. R: A language and environment for statistical computing. R Foundation for Statistical Computing, Vienna, Austria. URL https://www.R-project.org/. 2019.

6. Beirnaert C, Meysman P, Vu TN, et al. speaq 2.0: a complete workflow for high-throughput 1D NMR spectra processing and quantification. *PLoS Comput Biol*. 2018;14(3):1-23. http://biorxiv.org/content/early/2017/06/06/138503.abstract.

7. Southam AD, Weber RJM, Engel J, Jones MR, Viant MR. A complete workflow for high-resolution spectral-stitching nanoelectrospray direct-infusion mass-spectrometry-based metabolomics and lipidomics. *Nat Protoc*. 2017;12(2):255-273. doi:10.1038/nprot.2016.156

8. Dieterle F, Ross A, Schlotterbeck G, et al. Probabilistic Quotient Normalization as Robust Method to Account for Dilution of Complex Biological Mixtures. Application in 1 H NMR Metabonomics. *Anal Chem*. 2006;78(13):4281-4290. doi:10.1021/ac051632c

9. Hastie T, Tibshirani R, Sherlock G, Eisen M, Brown P, Botstein D. Imputing missing data for gene expression arrays. *Stanford Univ Stat Dep Tech Rep httpwwwstat stanford edu Hast pdf cll qxd*. 2006;3:27. http://citeseerx.ist.psu.edu/viewdoc/download?doi=10.1.1.79.9789&amp;rep=rep1&amp;type=pdf.

10. Parsons HM, Ludwig C, Günther UL, Viant MR. Improved classification accuracy in 1- and 2-dimensional NMR metabolomics data using the variance stabilising generalised logarithm transformation. *BMC Bioinformatics*. 2007;8(1):234. doi:10.1186/1471-2105-8-234

11. Rocke D, Lee GC, Tillinghast J, Durbin-Johnson B, Wu S. LMGene: LMGene Software for Data Transformation and Identification of Differentially Expressed Genes in Gene Expression Arrays. 2018.

12. Di Guida R, Engel J, Allwood JW, et al. Non-targeted UHPLC-MS metabolomic data processing methods: a comparative investigation of normalisation, missing value imputation, transformation and scaling. *Metabolomics*. 2016;12(5):1-14. doi:10.1007/s11306-016-1030-9

13. Wevers RA, Engelke U, Heerschap A. High-resolution 1H-NMR spectroscopy of blood plasma for metabolic studies. *Clin Chem*. 1994;40(7 I):1245-1250.

14. Rohart F, Gautier B, Singh A LCK-A. mixOmics : An R package for ‘ omics feature selection and multiple data integration. *PLoS Comput Biol*. 2017;13(11).

15. JOLLIFFE I. *Principal Component Analysis*. Vol 3.; 2005. doi:10.1201/b17700-1

16. Wold S, Sjöström M, Eriksson L. PLS-regression: A basic tool of chemometrics. *Chemom Intell Lab Syst*. 2001;58(2):109-130. doi:10.1016/S0169-7439(01)00155-1

17. Szymańska E, Saccenti E, Smilde AK, Westerhuis JA. Double-check: Validation of diagnostic statistics for PLS-DA models in metabolomics studies. *Metabolomics*. 2012;8:3-16. doi:10.1007/s11306-011-0330-3

18. Westerhuis JA, Hoefsloot HCJ, Smit S, et al. Assessment of PLSDA cross validation. *Metabolomics*. 2008;4(1):81-89. doi:10.1007/s11306-007-0099-6

19. Mehmood T, Liland KH, Snipen L, Sæbø S. A review of variable selection methods in Partial Least Squares Regression. *Chemom Intell Lab Syst*. 2012;118:62-69. doi:10.1016/j.chemolab.2012.07.010

20. Van Velzen EJJ, Westerhuis JA, Van Duynhoven JPM, et al. Multilevel data analysis of a crossover designed human nutritional intervention study. *J Proteome Res*. 2008;7(10):4483-4491. doi:10.1021/pr800145j

21. Nathan VanHoudnos. mclapply-hack.R. https://www.r-bloggers.com/implementing-mclapply-on-windows-a-primer-on-embarrassingly-parallel-computation-on-multicore-systems-with-r/. Published 2014.

22. The Digital Research Environment (DRE).

23. Brodersen KH, Ong CS, Stephan KE, Buhmann JM. The balanced accuracy and its posterior distribution. *Proc - Int Conf Pattern Recognit*. 2010:3121-3124. doi:10.1109/ICPR.2010.764

24. Abdi H. Partial least squares regression and projection on latent structure regression (PLS Regression). *Wiley Interdiscip Rev Comput Stat*. 2010;2(1):97-106. doi:10.1002/wics.51

25. Saccenti E, Hoefsloot HCJ, Smilde AK, Westerhuis JA, Hendriks MMWB. Reflections on univariate and multivariate analysis of metabolomics data. *Metabolomics*. 2014;10(3):361-374. doi:10.1007/s11306-013-0598-6

26. Royston P. Approximating the Shapiro-Wilk W-test for non-normality. *Stat Comput*. 1992;2(3):117-119. doi:10.1007/BF01891203

27. Bauer DF. Constructing confidence sets using rank statistics. *J Am Stat Assoc*. 1972;67(339):687-690. doi:10.1080/01621459.1972.10481279

28. Best DJ, Roberts DE. The Upper Tail Probabilities of Spearman ’ s Rho. *J R Stat Soc*. 1975;24(3):377-379.

29. Xu W, Hou Y, Hung YS, Zou Y. A comparative analysis of Spearmans rho and Kendalls tau in normal and contaminated normal models. *Signal Processing*. 2013;93(1):261-276. doi:10.1016/j.sigpro.2012.08.005

30. Erlic Z, Kurlbaum M, Deutschbein T, et al. Metabolic impact of pheochromocytoma/ paraganglioma: Targeted metabolomics in patients before and after tumor removal. *Eur J Endocrinol*. 2019;181(6):647-657. doi:10.1530/EJE-19-0589

31. Benjamini Y, Hochberg Y. Controlling the False Discovery Rate : A Practical and Powerful Approach to Multiple Testing. *J R Stat Soc*. 1995;57(1):289-300.

32. Team RC. R: A language and environment for statistical computing. R Foundation for Statistical Computing, Vienna, Austria. 2020. https://www.r-project.org/.

33. Wei T, Simko V. R package “corrplot”: Visualization of a Correlation Matrix. 2017. https://github.com/taiyun/corrplot.

34. Kassambara A. Correlation matrix : An R function to do all you need. http://www.sthda.com/english/wiki/correlation-matrix-an-r-function-to-do-all-you-need.
